# Evaluation of Alacepril Administration in Canine Patent Ductus Arteriosus According to Plasma Chymase Activity

**DOI:** 10.3390/ani14071078

**Published:** 2024-04-02

**Authors:** Kazumi Shimada, Miki Hirose, Lina Hamabe, Shinji Takai, Denan Jin, Zeki Yilmaz, Meric Kocaturk, Ryou Tanaka

**Affiliations:** 1 Department of Veterinary Medicine, Facility of Agriculture, Tokyo University of Agriculture and Technology, Fuchu 183-8509, Japan; kazumi-s@go.tuat.ac.jp (K.S.); s212240r@st.go.tuat.ac.jp (M.H.); ryo@vet.ne.jp (R.T.); 2 Department of Innovative Medicine, Graduate School of Medicine, Osaka Medical and Pharmaceutical University, Takatsuki-City 569-8686, Japan; shinji.takai@ompu.ac.jp (S.T.); denan.jin@ompu.ac.jp (D.J.); 3 Department of Internal Medicine, Faculty of Veterinary Medicine, Bursa Uludag University, Bursa 16059, Turkey; zyilmaz@uludag.edu.tr (Z.Y.); merick@uludag.edu.tr (M.K.)

**Keywords:** ACE inhibitor, chymase, PDA, antihypertensive action, dogs

## Abstract

**Simple Summary:**

Chymase is a protease stored in mast cell granules and is released triggered by tissue, especially cardiovascular, damage. Many studies showed the potentials of measuring chymase activity for the prognostic factor. However, the mechanism of chymase in veterinary cardiac disease was unknown. Recently, the plasma chymase activity has become possible to measure. Moreover, in patent ductus arteriosus, a congenital heart disease with a high incidence in veterinary medicine, chymase activity was significantly high at the preoperative time. In the present study, the changes of plasma chymase activity were further investigated after medical therapy for preoperative cardiac disease. The measurement of plasma chymase activity may be a useful tool for diagnosing the pathophysiology and the effect of medical therapy.

**Abstract:**

Chymase in the renin–angiotensin system (RAS) actively contributes to cardiac disease progression. Chymase is activated to produce angiotensin II during tissue injury and is involved in hemodynamics. A recent study demonstrated that plasma chymase activity reflects hemodynamic changes and aids in understanding patent ductus arteriosus (PDA) pathophysiology. The present study examined the relationship between plasma chymase activity and the administration of angiotensin-converting enzyme (ACE) inhibitor. Alacepril was administered to 13 puppies with PDA. Conventional echocardiographic parameters and non-invasive blood pressure were measured before and after medication. Plasma chymase activity was calculated using the colorimetric absorbance method. Plasma chymase activity significantly increased, but blood pressure significantly decreased. We detected an increase in plasma chymase activity due to ACE inhibition in PDA cases treated with alacepril. Plasma chymase activity was affected and altered by alacepril. In veterinary medicine, plasma chymase activity may be a novel method for assessing the pathology of and therapy for cardiac diseases.

## 1. Introduction

Chymase is one of serine proteases and, like angiotensin-converting enzyme (ACE), produces angiotensin II (ANGII). However, unlike ACE, chymase is characterized by its activation at the time of cardiovascular tissue injury [1]. Chymase is mainly present in mast cells, fibroblasts, and vascular endothelial cells [2], and is released into the extracellular matrix in response to inflammatory signals, tissue injury, or cellular stress [1]. Chymase has been reported to be involved in inflammation and tissue remodeling [3,4]. In dogs, chymase accounts for nearly 90% of ANGII production in the heart [5], while ACE contributes about 70% in blood vessels [6]. Additionally, chymase produces ANGII and activates MMP-9, which is involved in inflammation and TGF-β in the fibrosis of cardiovascular tissue [7,8]. Due to these characteristics, chymase inhibition has been studied as a medication that focuses specifically on injury, unlike ACE inhibitors. Chymase research in recent years has focused on human diseases related to the cardiovascular system. For example, a chymase inhibitor was administered to myocardial infarction patients in human clinical trials [9], and the effects of chymase inhibition were studied using thrombotic model mice [10]. Chymase inhibition has not yet been reported in clinical veterinary medicine.

In clinical veterinary medicine, measuring chymase activity is primarily challenging regarding using chymase as a therapeutic target. Although measurement has generally been based on tissue sampling, it is unsuitable for clinical application. Wang et al. reported increased chymase activity from human plasma in hypertensive cases [2]. Although this report demonstrates the measurement of plasma chymase activity, few reports have been observed in veterinary medicine. The second challenge is that almost no chymase studies focused on veterinary medicine. The previous reports using animal models target common diseases in human medicine, such as myocardial infarction [3] and arrhythmic heart failure [11]. Some chymase studies in veterinary medicine have been conducted in dog models of mitral valve disease as a capacitance-loading disease. However, the pathogenesis of the disease differed from the original pathophysiology [7]. Due to these research backgrounds, the clinical application of chymase has not progressed in veterinary medicine.

Patent ductus arteriosus (PDA), a congenital heart disease, involves post-birth persistence of ductus arteriosus, which is necessary for systemic circulation during fetal life. Although PDA has a high incidence in dogs [12,13,14], the prognosis is considered good after surgical treatment [15]. In early PDA, blood flows from the aorta into the pulmonary artery and back again through the lungs to the left atrium. Therefore, it is necessary to treat PDA and prevent progression to congestive heart failure as a capacitance load disease [16,17]. Our previous study showed that plasma chymase activity significantly decreased after surgical treatment in PDA puppies [18]. This result indicates that plasma chymase activity reflects changes in hemodynamics. Therefore, the utility of plasma chymase activity has been suggested to evaluate the pathogenesis of cardiac disease in veterinary medicine.

Further studies are needed to evaluate the pathogenesis of cardiac disease using plasma chymase activity. While the efficacy of surgical treatment for PDA has already been demonstrated by plasma chymase activity, it is unclear how it is altered by medical treatment, especially drug administration. In the report by Su et al., ACE inhibitors were administered to mitral valve disease dog models, and chymase activity was measured using cardiac tissue [19]. The present study focuses on the use of ACE inhibitors to suppress hyperactivation of the renin–angiotensin system (RAS) in the management of PDA. This study hypothesized that ACE pathway inhibition caused an increase in plasma chymase activity in PDA. Herein, PDA puppies were treated with alacepril, and comparisons were made of blood pressure, echocardiography, and plasma chymase activity before and after treatment.

## 2. Materials and Methods

### 2.1. Animals and Medication

Puppies with cardiac murmur were brought to the Dog and Cat Pediatric Hospital for diagnosis. After informed consent was obtained from all owners, the puppies underwent echocardiography by using a 14–3 MHz phased array transducer probe (Lisendo 880LE and S42; FUJIFILM, Tokyo, Japan), blood pressure measurement, and blood testing according to the method of our previous study [17]. The diagnosis of PDA was confirmed through echocardiography at the right short-axis view by using color Doppler. Echocardiography parameters were collected, including the PDA diameter, fractional shortening (FS%), early diastolic left ventricular inflow velocity (E vel), systolic (s’) and early diastolic (e’) mitral ring velocity at the septal mitral annulus (Septal), and left ventricular free wall (LVFW).

Non-invasive blood pressure measurements (systolic, diastolic, mean) were conducted using the oscillometric method (Pet MAP graphic II; ManoMedical, Taden, France).

Alacepril was administered at a dose of 3.64 ± 0.76 mg/kg/day (BID, Apinac©, DS Pharma Animal Health, Osaka, Japan).

### 2.2. Plasma Sample and Measurement of Plasma Chymase Activity

Blood was sampled from the vein and anticoagulated with heparin due to preoperative blood testing. After centrifugation (4000 rpm, 5 min) and blood testing, excess plasma was frozen. About 5 days after medication, echocardiography and blood tests were performed and excess plasma was also stored in the freezer. Chymase activity was measured according to the method of our previous study [17]. Plasma (40 μL) was mixed with Tris buffer (40 μL; 100 mM Tris and 1 M NaCl at pH 8.0, Wako Pure Chemical Industries, Ltd., Osaka, Japan). Dimethyl sulfoxide (1 μL; DMSO, Nacalai Tesque, Inc., Kyoto, Japan) was added to the control well, and 1 μL of chymase inhibitor (10 mM Suc-Val-Pro-Phe^p(0Ph)2, Peptide Institute Inc., Osaka, Japan) was added to the other well. After one hour at room temperature, 1 μL of chymase substrate (50 mM Suc-Val-Ala-Ala-Pro-Phe-pNA in DMSO, Peptide Institute Inc., Osaka, Japan) was added to both wells and mixed for 5 min at room temperature. After 1 and 2 h of incubation at 37 °C, the plate was read at 405 mm with a microplate reader (INFINITE 200 PRO, Tecan Austria GmbH, Grödig, Austria). The unit of chymase activity, U, was used as μmol/min pNA.

### 2.3. Statistical Analysis

The age at blood sampling before and after medication were counted as median days from birth (minimum–maximum). Body weight, echocardiographic parameters, blood pressure, and chymase activity are presented as the mean ± SD. Statistical analysis was performed by using software (Graph Pad Prism 8, MDF Co., Ltd., Tokyo, Japan). A paired *t*-test was used for chymase activity and echocardiography parameters. The non-parametric Mann–Whitney U test was used for blood pressure. *p* < 0.05 was considered statistically significant.

## 3. Results

### 3.1. Animals and Medication

Thirteen puppies, including 4 Pomeranians, 3 toy mix-breeds, 2 miniature dachshunds, 2 toy poodles, 1 Shetland sheepdog, and 1 Maltese, were diagnosed with PDA, and the evaluation of chymase activity was performed (Table 1). The puppies did not present with severe PDA, congestive heart failure requiring immediate surgery, or pulmonary hypertension. Alacepril was administered to the 3 males and 10 females. Some cases required treatment with diuretics and antibiotics due to signs of congestion; cases needed to maintain their clinical condition before surgery. At the time of initial presentation (before-medication), they were 63 (59–143) days of age and weighed 0.65 ± 0.4 kg. After medication, they were 70 (63–147) days of age and weighed 0.86 ± 0.41 kg.

### 3.2. Echocardiography Parameters and Non-Invarsive Blood Pressure

**Figure 1:**
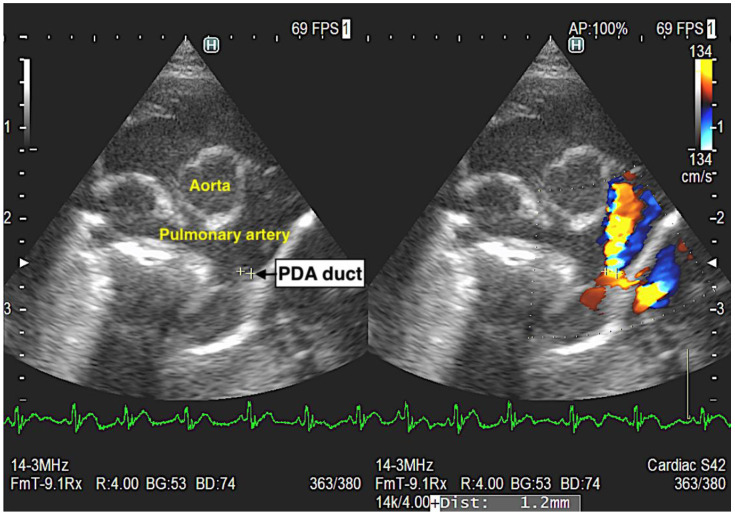
Echocardiographic image of PDA duct and PDA blood flow. All cases showed the left–right sunt blood flow.

Table 2 presents echocardiographic parameters and blood pressure. The diameter of PDA was 1.9 ± 0.45 mm (Figure 1). Echocardiographic parameters showed no significant differences before or after medication. Eight cases were excluded from the blood pressure data because they were of very small size, making measurement difficult using the oscillometric method. Significant differences were observed in the systolic, mean, and diastolic blood pressure (*p* = 0.009, *p* = 0.007, and *p* = 0.0001, respectively).

### 3.3. Plasma Chymase Activity

**Figure 2:**
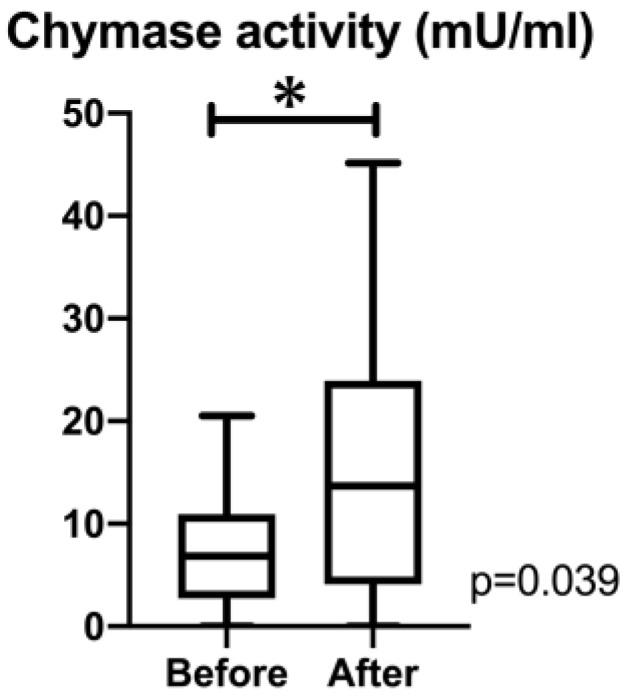
Chymase activity before and after the administration of alacepril. Plasma chymase activity increased significantly after the administration of alacepril. *, *p* < 0.05.

Figure 2 shows the plasma chymase activity, which increased significantly after medication (*p* = 0.039).

## 4. Discussion

Changes in plasma chymase activity were investigated to assess the pathogenesis of congenital cardiac disease. In the present study, alacepril, an ACE inhibitor, was administered for the preoperative management of PDA. Blood pressure, echocardiographic parameters, and plasma chymase activity were compared before and after medication. Blood pressure significantly decreased with the administration of alacepril. This result is attributed to the antihypertensive effect of alacepril [20,21], which has prevented an increase in afterload as an ACE inhibitor. In PDA cases with hypertension, the antihypertensive effect of alacepril was suggested to be useful. Echocardiography showed no significant difference before and after medication. FS and s’, indicating cardiac contractility, were not decreased; therefore, the results ruled out an adverse event caused by alacepril. E vel and E/e’, which are indicators of congestion and evaluate capacity load, did not change after alacepril administration. Since PDA is a capacity load disease and these indexes are expected to worsen with progression, the worsening PDA pathology was negative during the present study. From these results, although alacepril administration did not exactly improve cardiac function, PDA pathology was not worsening due to treatment with alacepril.

**Figure 3:**
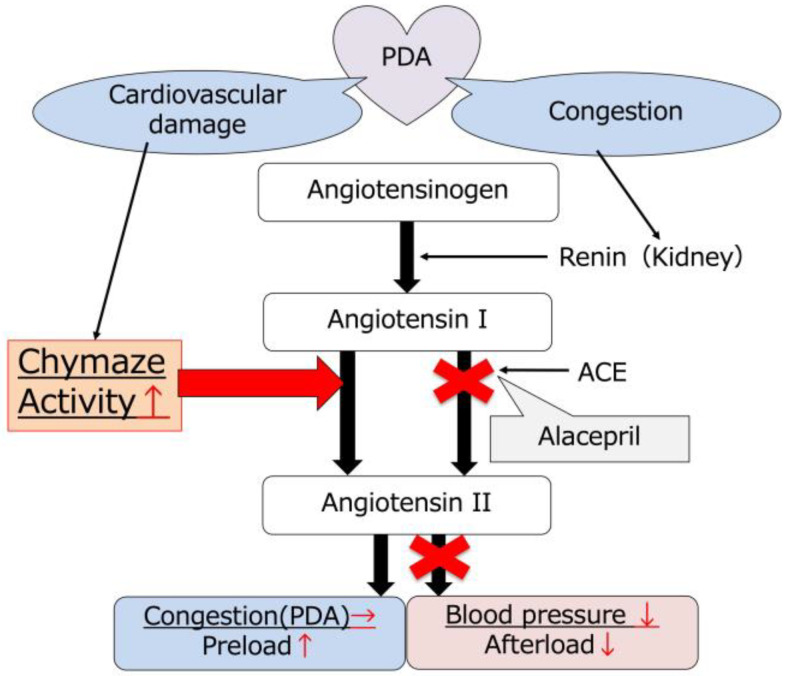
Schema of the present study. Alacepril inhibited the ACE-dependent pathway and decreased afterload due to improved hypertrophy. Plasma chymase activity increased due to decreased angiotensin II after the administration of alacepril.

Plasma chymase activity significantly increased after the administration of alacepril. The reaction was considered to be caused by cardiovascular damage due to the progression of PDA pathology and/or a decrease in ANGII produced from the ACE pathway (Figure 3). The present study suggests that reducing the afterload by decreasing blood pressure and avoiding worsening cardiac function and congestion may minimize cardiovascular damage from PDA progression. However, this discussion is limited by the fact that histological examination is required to evaluate these disorders, and clinical examination alone is inadequate. On the other hand, the present study showed interesting results regarding ANGII production by chymase and ACE. PDA cases in the present study showed hypertension, most probably due to elevated ANGII production to prevent the homeostasis of RAS. The decrease in ANGII due to ACE inhibition may have led to an exaggerated response of chymase activity. Therefore, the plasma chymase activity results were consistent with our hypothesis and may represent a compensatory response of RAS to the inhibition of the ACE-dependent pathway. Plasma chymase activity was suggested to have been changed by medical treatment, and it might be a reasonable parameter for the assessment of medication efficacy. However, a previous report has described that chymase-derived ANGII production is positively correlated with systolic blood pressure [22]. Su et al. reported that ramipril, an ACE inhibitor, was administered to dogs with mitral regurgitation, a volume overload disease similar to PDA; this increased chymase activity in cardiac tissue compared with normal dogs, with or without ACE inhibition [19]. Although the present study confirmed the medication response at short-term until surgical intervention, these reports would also predict different results of chymase activity and blood pressure in long-term alacepril administration or chronic cardiac disease. Comparisons with PDA cases without ACE inhibitors and changes in ACE activity and ANGII production require further investigation.

Although ACE and chymase activities were reported in dogs and cats [23], the relationship between cardiac tissue and plasma chymase activity has not yet been examined. Whether plasma chymase activity is as informative is debatable. The present results suggest that plasma chymase activity may be influenced by blood pressure, which is a function of blood vessels, rather than by contractility, which is strongly influenced by cardiac function, especially myocardial tissue. An invasive study, such as sampling cardiac tissue to use for histopathology and measuring tissue chymase, is urgently needed to detect myocardial tissue damage and compare with plasma chymase to establish the use of plasma chymase as a biomarker.

In the present study, the administration of alacepril caused an antihypertensive effect and increased plasma chymase activity in PDA cases. As a part of the preoperative management of PDA, the administration of ACE inhibitors may activate chymase, contributing to fibrosis and inflammation in the myocardium. Since PDA cases in the present study showed hypertension without causing any cardiac dysfunction, the hypotensive effect from alacepril may have been useful in maintaining the preoperative condition. However, the possibility of worsening pathology due to increased chymase activity suggests that surgical treatment should be performed in PDA when plasma chymase activity is elevated. During the preoperative management of PDA, appropriate ACE inhibitors such as alacepril should be selected based on blood pressure and cardiac function in each case, considering the upregulation of plasma chymase activity. Considering that the RAS mechanism is very complex, the present study has limitations in completely understanding the chymase pathway in PDA due to the unavailability of clinical data, small sample size, and no control data. Even with this limitation, this study’s findings on plasma chymase activity are expected to provide valuable information for veterinarians regarding whether drugs such as chymase inhibitors should be used in combination. The measurement of plasma chymase activity may allow for appropriate RAS pathway inhibition to control hypertension and heart failure.

Future studies are expected to compare how plasma chymase activity changes with the administration of other ACE inhibitors or cardiovascular agonists, and utilize chymase activity to select appropriate medical treatment. The measurement of plasma chymase activity is expected to be a valuable parameter for cardiovascular diseases treated with medication.

## 5. Conclusions

In this study, we observed an increase in plasma chymase activity after ACE inhibition in PDA cases treated with alacepril. Plasma chymase activity was affected and altered by RAS. In veterinary medicine, plasma chymase activity could be applied as a novel pathological and therapeutic assessment method in cardiac diseases.

## Figures and Tables

**Table 1:** Pappies with PDA signalment.

**Breed**	**Number (*n* = 13)**
Pomeranian	4
Toy mix-breed	3
Miniature dachshund	2
Toy poodle	2
Shetland sheepdog	1
Maltese	1
**Gender**	**Number (*n* = 13)**
Female	10
Male	3

**Table 2:** Echocardiography parameters and blood pressure before and after the administration of alacepril.

Echocardiography Parameter	Before	After	*p*-Value
FS (%)	41.4 ± 7.85	38.5 ± 8.47	0.13
E vel (cm/s)	103.3 ± 10.27	113.5 ± 17.06	0.37
Septal s’ (cm/s)	5.1 ± 1.99	6.85 ± 1.69	0.13
Septal e’ (cm/s)	7.4 ± 2.92	8.1 ± 2.35	0.77
Septal E/e’	12 ± 2.65	11.51 ± 2.71	0.73
LVFW s’ (cm/s)	6.4 ± 2.9	6.5 ± 1.2	0.89
LVFW e’ (cm/s)	9.3 ± 3.05	10.3 ± 2.61	1.0
LVFW E/e’	9.84 ± 2.49	8.6 ± 2.23	0.71
**NIBP**	**Before**	**After**	***p* value**
Systolic	192 ± 35.28	143 ± 42.94 **	0.009
Mean	125 ± 33.92	109 ± 26.72 **	0.007
Diastolic	108 ± 26.01	77.5 ± 22.57 ***	0.0001

FS, fractional shortening; E vel, early diastolic left ventricular inflow velocity; Septal, septal mitral annulus; LVFW, left ventricular free wall; s’, systolic mitral ring velocity; e’, early diastolic mitral ring velocity; NIBP, non-invasive blood pressure. **, *p* < 0.01; ***, *p* < 0.001.

## Data Availability

The original contributions presented in the study are included in the article, further inquiries can be directed to the corresponding author.
